# A Survey on the Computing Continuum and Meta-Operating Systems: Perspectives, Architectures, Outcomes, and Open Challenges

**DOI:** 10.3390/s26030799

**Published:** 2026-01-25

**Authors:** Panagiotis K. Gkonis, Anastasios Giannopoulos, Nikolaos Nomikos, Lambros Sarakis, Vasileios Nikolakakis, Gerasimos Patsourakis, Panagiotis Trakadas

**Affiliations:** 1 Department of Digital Industry Technologies, National and Kapodistrian University of Athens, Evripus Campus, 34400 Euboea, Greece; lsarakis@uoa.gr; 2 Department of Ports Management and Shipping, National and Kapodistrian University of Athens, Evripus Campus, 34400 Euboea, Greece; angianno@uoa.gr (A.G.); vnikolak@uoa.gr (V.N.); gerasimos.patsourakis@gmail.com (G.P.); ptrakadas@uoa.gr (P.T.); 3 Department of Information and Communication Systems Engineering, University of the Aegean, 83200 Samos, Greece; nnomikos@aegean.gr

**Keywords:** Meta-Operating Systems, cloud continuum, machine learning, Internet of Things, security and privacy

## Abstract

The goal of the study presented in this work is to analyze all recent advances in the context of the computing continuum and meta-operating systems (meta-OSs). The term continuum includes a variety of diverse hardware and computing elements, as well as network protocols, ranging from lightweight Internet of Things (IoT) components to more complex edge or cloud servers. To this end, the rapid penetration of IoT technology in modern-era networks, along with associated applications, poses new challenges towards efficient application deployment over heterogeneous network infrastructures. These challenges involve, among others, the interconnection of a vast number of IoT devices and protocols, proper resource management, and threat protection and privacy preservation. Hence, unified access mechanisms, data management policies, and security protocols are required across the continuum to support the vision of seamless connectivity and diverse device integration. This task becomes even more important as discussions on sixth generation (6G) networks are already taking place, which they are envisaged to coexist with IoT applications. Therefore, in this work the most significant technological approaches to satisfy the aforementioned challenges and requirements are presented and analyzed. To this end, a proposed architectural approach is also presented and discussed, which takes into consideration all key players and components in the continuum. In the same context, indicative use cases and scenarios that are leveraged from a meta-OSs in the computing continuum are presented as well. Finally, open issues and related challenges are also discussed.

## 1. Introduction

The rapid growth of the number of interconnected devices on the internet (Internet of Things—IoT) has posed new challenges for the design and implementation of flexible network architectures that can handle both a vast number of IoT components (i.e., proper access and resource-management protocols) and associated security threats [1,2]. In the early years of IoT technology, data generated in the IoT devices was forwarded directly to the cloud domain via classical network infrastructure. However, this strategy can impose certain limitations, mainly related to the increased round trip time in cases of delay-sensitive and latency-critical applications. Moreover, technological advances in the IoT domain have made feasible the deployment of advanced services and applications that do not simply rely on data gathering and processing from the IoT network but actively participate in process optimization. Typical examples include automation sensors in industrial 4.0 scenarios, as well as smart building sensors. Hence, the need for reduced latency is inextricably combined with the ability to perform time-consuming calculations at the edge of the network [3,4].

Over the last decade, a new architectural approach has emerged, leveraging the so-called Cloud-Edge-IoT (CEI) paradigm [5,6]. In this context, edge servers located near the IoT devices process related data and perform time-consuming calculations, depending on their computational capacity. The outcomes of these calculations (i.e., encryption procedures, optimization of key performance indicators, training of a machine learning—ML model, or creation of blockchains) are sent directly to the IoT devices, without the need for central cloud processing and long round-trip times. To this end, the cloud domain either receives corresponding results at a later stage, or it is directly involved in cases of high-complexity calculations. Hence, mean response times can be significantly reduced with respect to centralized cloud domain processing. All devices that constitute this complex IoT, edge and cloud ecosystem, are also referred to as the computing continuum. In comparison to single vendor clouds or hybrid clouds centrally managed, heterogeneous IoT devices, data, network, edge, and cloud components are significantly more complex to manage, since the continuum integrates a variety of diverse hardware and software elements. To this end, the addition of new devices and protocols can be a scalability-challenging task. The continuum needs to be efficiently managed to optimally meet the application demands during service execution, by not only bringing computation closer to where the data is produced, but also by placing and formatting the data to optimize the execution, both for real-time services and non-real time data analytics [7,8].

**Figure 1:**
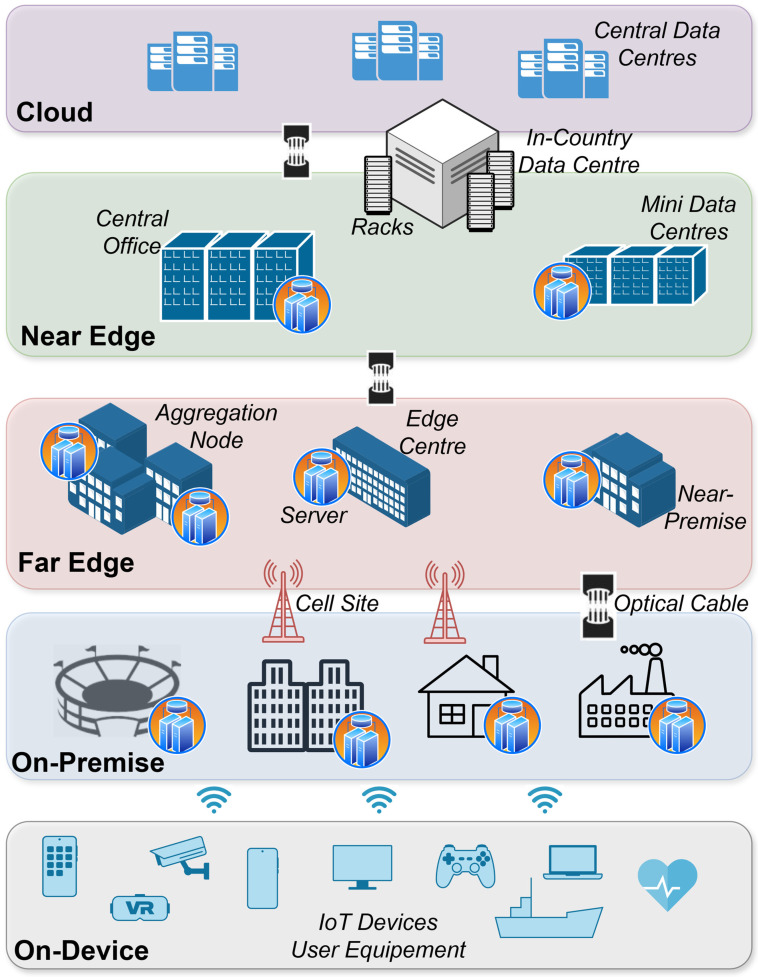
The Cloud-Edge-IoT concept disaggregated in five computing tiers.

In this context, a high-level view of the CEI concept is depicted in Figure 1. The lower layer includes IoT devices, as well as user equipment. All these items generate a vast amount of heterogeneous data that needs to be stored and processed accordingly in a secure and privacy-preserving way. Moving a step forward, the next layer includes the on-premise layer. To this end, processing nodes with improved computational capacity are located within the premise, e.g., a stadium, an enterprise, or a smart home. This can be highly beneficial in industrial scenarios, for example, where on one hand, sensitive data remains within the facilities of the enterprise and, on the other hand, latency times can be significantly improved. In the far and near-edge layer, high computing nodes that are in close or far proximity to the cloud servers gather data and train advanced ML algorithms and, in general, perform time-consuming calculations. In this context, certain tasks that require high computational power can be offloaded from the on-premise servers. In the cloud domain, a two-fold process is carried out: (a) ML model aggregation and update from the individual models that were constructed in the previous layers and (b) large-scale data processing that was not made feasible in the previous steps due to limited resources.

As the concept of CEI systems is gradually developing, various research efforts have dealt with operating systems and policy configurations that span across this continuum. To this end, diverse challenges are dealt with, such as access to the continuum, the flexible usage of resources and optimum resource management, performance improvement, threat prediction and mitigation, etc. [9,10]. In the same context, intelligent edge computing and ML are also two promising approaches that can leverage the deployment of the continuum in lightweight devices [11,12]. All the aforementioned topics may represent highly demanding tasks, not only due to the continuum being intrinsically heterogeneous, volatile, distributed, and increasingly cognitive, but also due to emerging requests to be open und collaborative. A holistic approach towards the solutioning of this technological trend in future systems can be achieved by architecting, designing, and implementing the continuum as extensible, open, secure, adaptable, and artificial intelligence (AI)-powered, as well as highly performant and technology-agnostic.

**Figure 2:**
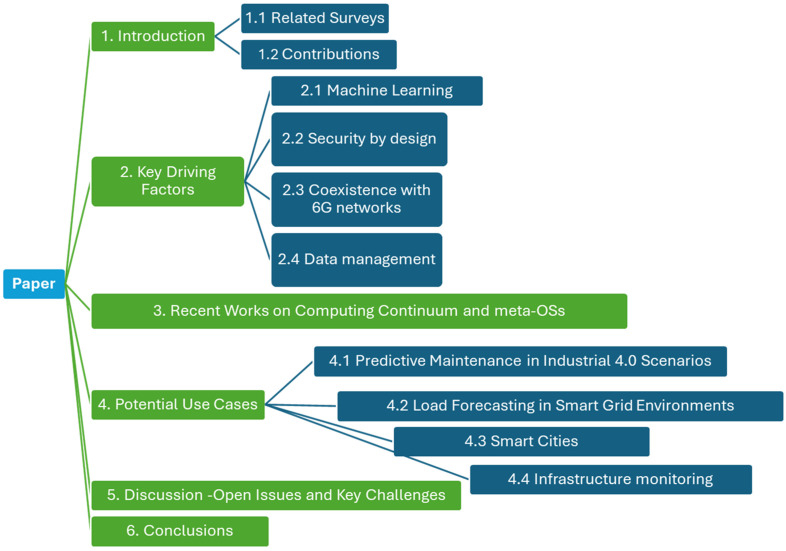
Structure of the present work.

The goal of the survey presented in this work is to analyze all recent developments in the context of meta-OSs and the computing continuum. In this context, various recent works are presented and analyzed, with respect to the aforementioned challenges and proposed technological solutions. Moving forward, a high-level architectural view of a meta-OS system is presented as well, along with indicative use cases and scenarios. A schematic overview of the structure of our work is depicted in Figure 2, for illustration purposes.

### 1.1. Related Surveys

In this subsection, indicative recent related survey papers are presented with respect to their key outcomes. Afterwards, the main contributions of our work are highlighted. To this end, in [13] a survey is provided for cloud-edge workload orchestration at the edge. The orchestration of workloads can be a quite challenging task, due to the heterogeneity of computing equipment in edge scenarios. As the authors correctly point out, a potential solution lies in the creation of lightweight versions of Kubernetes. Another promising approach is the use of KubeEdge [14], which is specifically designed for edge applications, instead of trying to reduce the size of Kubernetes. However, as the authors indicate, containers can have certain disadvantages that are mainly associated with a large image size and non-robust security. Hence, an alternate approach involves the use of hardware-level or lightweight virtualization mechanisms, such as virtual machines (VMs) or MicroVMs, which can provide stronger isolation guarantees compared to container-based OS-level virtualization, particularly in heterogeneous and security-sensitive edge environments.

In [5], a survey on CEI systems is provided, which focuses on key enabling technologies, as well as on the presentation of all recent works on the field. In the same context, limitations and open challenges are discussed as well. To this end, indicative use cases are also presented that are leveraged from the CEI concept. In [15], all latest architectural approaches in computing continuum systems are described. In particular, this work focuses on AI for applications deployed at the edge, as well as for efficient AI techniques to manage the workload in the edge of the computing continuum.

In [16], the general architecture of distributed computing systems (DCSs) is presented. In the same context, the authors describe various potential applications, as well, that are based on DCSs. These include, among others, industry automation, transportation systems, mobile robots, smart cities, and health care. In [17], this survey paper analyzes all recent trends of computing continuum systems. In this regard, potential use cases include highly mobile self-driving vehicles, holographic streaming services (Telepresence), ultra-reliable industrial IoT, and urgent computing. In the same context, various open issues are also discussed, that include, among others, proper resource allocation and management, simulation tools for large scale performance evaluation, the integration of mobility in the continuum, and programming distributed applications.

Finally, the survey in [18] focuses on the applications of software-defined networking (SDN), as well as network function virtualization (NFV), in the management, orchestration, and load balancing of workloads in cloud/edge orientations. Various challenges are also identified, such as distributed architectures, dynamic offloading of resources, security and privacy, intelligent orchestration, and work management. The key outcomes of each survey presented are also depicted in Table 1, where open issues and limitations are highlighted as well. To this end, the main points of our work are indicated as well.

### 1.2. Contributions of This Work

In the works that were presented in the previous subsection, emphasis was placed on different parts of the cloud computing continuum (i.e., AI/ML deployments, containerization, virtualization, CEI and DCC architectures, NFV and SDN solutions, etc.). Therefore, unlike other similar works in the literature, our work tries to cover all individual aspects in the context of meta-OSs in the computing continuum. These include, among others, the presentation of the most important key enabling technologies, as well as all related research efforts in data management, security, and privacy, as well as resource optimization via ML. To this end, emphasis is given on the analysis of various European Union (EU)-funded projects. Therefore, the main contributions of our work can be summarized as follows:

- Investigation into all recent trends in architectural approaches regarding the integration of meta-OSs with the computing continuum.
- Emphasis on key enabling technologies such as security by design, coexistence with 6G networks, data management, and advanced AI/ML approaches that leverage response times and provide optimum resource management.
- A reference architectural approach is presented that takes into account all major players in the computing continuum and their interactions.
- Indicative use cases are presented, as well, that benefit from the cloud computing continuum.
- Finally, open issues are also identified to trigger further research activities.

## 2. Key Driving Factors

Meta-OSs and the computing continuum face numerous challenges that need to be dealt with, as described in Section 1. The interconnection of numerous diverse devices, on one hand, necessitates common access and data-management protocols and, on the other hand, brings to front more robust security and privacy-protection mechanisms, since the attack landscape is now significantly increased compared to previous generations of wireless networks. In the same context, resource management can be now a highly complex task, due to the vast number of heterogeneous devices and protocols. Moreover, the convergence of IoT devices, as well as edge and cloud servers is expected to leverage the deployment of advanced services and applications in 6G networks. To this end, various key enabling factors can be identified, such as efficient AI/ML approaches for proper resource optimization, as well as security and privacy protection, security by design, coexistence with 6G networks, and data management.

### 2.1. Machine Learning

A challenging research field in the design of the computing continuum systems is the use of appropriate ML approaches both for threat prediction and mitigation, as well as for resource optimization. In the latter case, the goal is to select the appropriate architectural layer (i.e., IoT, edge, cloud) and corresponding device where a certain task will be executed. This decision is based on numerous factors, such as task complexity, delay tolerance, energy consumption, etc. Hence, to this end, an efficient ML approach that can adapt to dynamic network orientations is deep reinforcement learning (DRL) [19,20]. In this context, a mobile agent interacts with the environment and selects the appropriate action for the task under consideration, according to a policy that maximizes the overall reward. Depending on the outcomes of this action, either positive or negative rewards are given to the mobile agent. After a sufficient number of training rounds, near-optimum decision goals can be achieved with the help of neural networks (NNs) that can be trained according to various pairs of potential states and actions. In this context, a quite popular approach is Q-learning, where the agent keeps a score of quality values that are updated after each system transition. To this end, the new q-value is based on the previous q-value and the received reward properly weighted.

**Figure 3:**
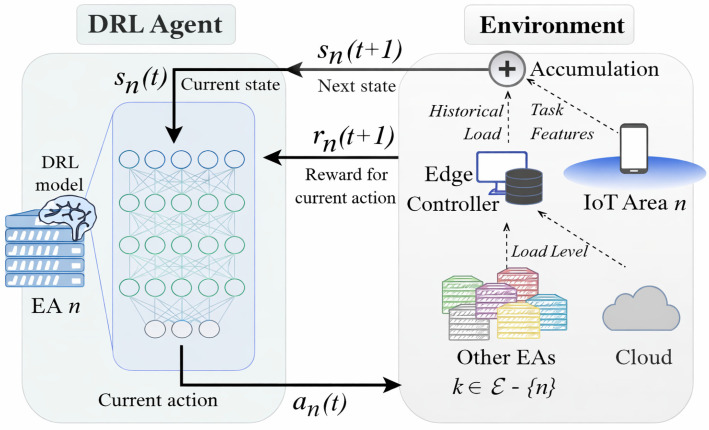
Deep reinforcement learning approach in Cloud-Edge-IoT systems for a given agent n∈ε. EA: edge agent; ε is the set of DRL edge agents.

**Figure 4:**
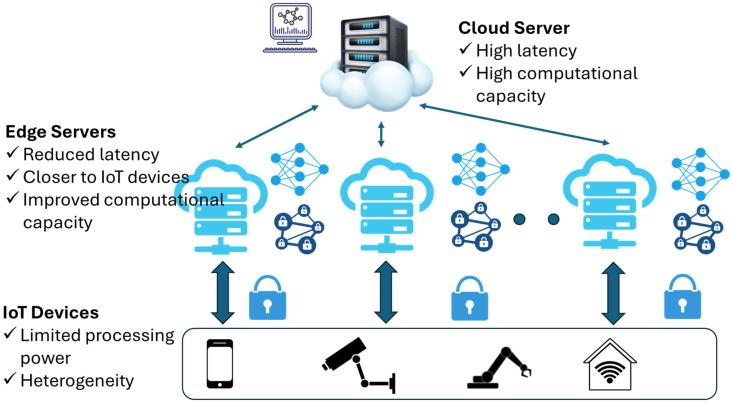
Federated Learning in the computing continuum with local training steps performed at edge layers and model aggregation implemented at the cloud.

A schematic overview of this approach is depicted in Figure 3. To this end, various data are gathered from the IoT devices, and the edge controllers and the cloud and are sent to the DRL agent. These data are related to distinct operational factors, such as the historical load, task features, and current load in the participating devices. The aforementioned approach can be easily applied in Federated Learning (FL) scenarios as well [21]. As depicted in Figure 4, multiple participating nodes that represent either IoT, edge, or cloud domains train an ML model locally, based on the data collected from their surrounding environment. Afterwards, ML model parameters, such as weights in the case of NN training, are send to the master ML server for model aggregation and updating. At this stage, model inference can take place as well. The master server, after processing and aggregating all parameters, sends the updated weights to the participating nodes. Therefore, on one hand, the computational burden is divided among the participating nodes, and, on the other hand, no sensitive data is transmitted. Hence, privacy-sensitive applications, such as e-health, can now be deployed more easily.

### 2.2. Security by Design

Another challenging research field in the management of the computing continuum is the provision of security by design during application execution. Since this continuum integrates various resource-constrained IoT devices, not all of them have the capability to execute advanced security protocols. Hence, various associated security challenges can be identified, such as protecting against unauthorized access, ensuring data security and isolation in a multitenant environment, and securing virtual machines (VMs) [22,23].

The use of virtualized network elements, open interfaces, and disaggregation that are extremely important in the cloud computing era can pose several security challenges. Unlike for example fifth-generation (5G) networks, where security solutions across all devices and base stations are configured with universal settings for certain types of attacks, it is apparent that such an approach cannot be applied in an integrated CEI infrastructure. In this case, the high diversity in service and application provision, connected devices, and associated protocols in heterogeneous networks, as well as the various physical-layer encoding and transmission techniques, render a highly complex environment with different requirements and settings. Since each scenario may have unique security requirements and energy availability, the selection and configuration of security strategies need to be optimized in an adaptive and dynamic manner. As it will be also discussed in the following subsection, the cloud computing continuum is envisaged to coexist with 6G networks to support advanced services and applications. The security attacks in 6G networks are polymorphic in nature and sophisticated, using previously unseen custom code, able to communicate with an external command and control entities to update their functionality or even implement themselves entirely from code fragments that they intelligently harvest from benign programs, scripts, and software blocks already present in the security system in place [24,25]. Therefore, on one hand, it is important to have strong security mechanisms in the computing continuum domain, such as the zero trust concept [26], and, on the other hand, to effectively design ML approaches that can create the appropriate intents in due time for a variety of potential attacks.

### 2.3. Coexistence with 6G Networks

It is expected that the 6G concept will be based on a holistic integration of broadband networks and lightweight IoT devices that can leverage various cutting-edge applications in the real world. Hence, the underlying infrastructure of the computing continuum, as well as meta-OSs, should be able to adapt to 6G network protocols and, in particular, the ones that are related to proper resource management and security. Typical advanced applications include, among others, autonomous driving, where high-precision sensors are mounted on vehicles, e-health applications with wearable sensors, and IoT devices, as well as industrial robotic applications [27,28]. In the same context, another promising novelty of 6G networks will be the subnetwork concept, where a network component in the edge acts as a serving access point (AP) in the case where connection with the main core network is lost [29,30]. Therefore, in this case, efficient task offloading and reduced latency computations in the edge/cloud domain are of utmost importance, to support critical applications. Moreover, as also referred to in the previous subsection, in the case of 6G networks, an increased attack landscape should now be dealt with. Hence, efficient network monitoring is required for threat prediction and mitigation via the creation of appropriate intents.

Finally, in the framework of 6G networks, a promising architectural approach that has emerged over the last years is the organic concept [31]. To this end, the long-term vision is to support the ability of 6G networks to dynamically adapt their resources according to user needs and traffic demands, within the available infrastructure resources. Hence, more flexible resource management can be supported, by leveraging the well-known concept of service-based architecture (SBA) of 5G networks. Hence, the meta-OS in the computing continuum should provide the appropriate mechanisms for dynamic device onboarding and detachment from different segments of the network, flexible data gathering, and ML model adjustment.

### 2.4. Data Management

An efficient data-management solution should address the requirements of heterogeneous and changing infrastructures by supporting dynamic and flexible data federation between devices, enabling the integration of data from independent and volatile sources within a single application. It will also enable the execution of parts of a service within the data platform to increase performance and favor privacy and will facilitate the development of services to be deployed on the continuum by abstracting data distribution, communication, and management details across the different layers of the infrastructure.

In [32] for example, a Message Queuing Telemetry Transport (MQTT) broker has been used for the communication of devices in the CEI continuum. The key features of MQTT are minimal network bandwidth usage, efficient message delivery, and support for a range of quality of service (QoS) levels to ensure message reliability. As the authors point out, although MQTT helps in reducing system vulnerabilities and facilitating compliance with data privacy requirements, there are still various open issues to be addressed. These include integration with emerging technologies, such as FL, DevOps, and more adaptive security policies for dynamic IoT environments.

Another important issue that should also be considered in the design of an efficient data-management system is legislative measures governing data sharing and privacy [33]. Hence, data-protection laws should be considered in the new ecosystem via the integration of modules that enable compliance with data-protection regulations while transferring the data across the continuum.

**Figure 5:**
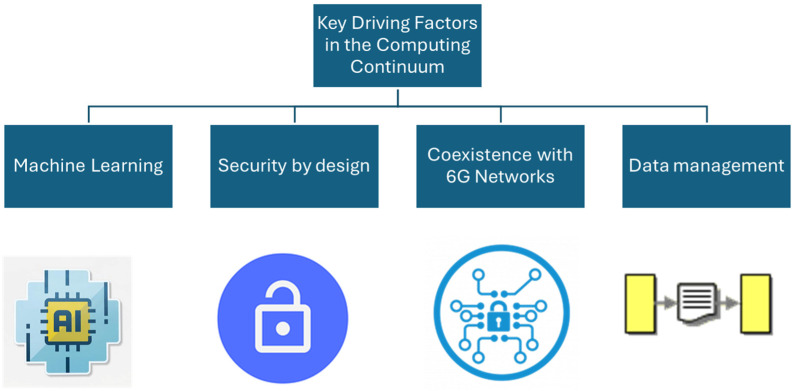
Key driving factors in the computing continuum.

A schematic overview of the most important key driving factors in the design of computing continuum systems is also depicted in Figure 5, for illustration purposes.

## 3. Recent Works on Computing Continuum and Meta-OSs

In this section, various recent works in the context of meta-OSs and the computing continuum are discussed. In [34], and in the context of the EU-funded project FLUIDOS [35], an AI driven approach is presented, to optimize resource allocation during application execution. To this end, a user may ask for the execution of a specific workflow, which is passed by the Operator API to the corresponding ML component. This component is trained using previous experience from similar user requirements. Consequently, this workload is translated to an equivalent set of resources to be committed during service execution. Performance evaluation took place with the help of two well-known datasets, and results were compared against the baseline approach (brute-force). In all cases, resource-allocation times were significantly reduced. In particular, two model architectures were considered: in the first case (Candidate Generation Model Architecture), a potential list of configurations is generated based on previous experience. In the second case (two-tower model architecture), an initial set of resources is initially extracted that leads to optimum selection based on a ranking model.

In [36], the NebulOuS architectural vision is presented and described, for secure and optimal application provisioning [37]. In this context, a model-driven approach is presented, where a user first describes an application and the required resources using a well-defined application model. In the same context, admin users also advertise their resources using NebulOuS. Hence, the continuum also acts as a gateway where different entities make use of heterogeneous resources. AI-driven threat detection and mitigation are also supported. In [38], the work presented by the authors is based on the EU-funded project NEMO [39]. To this end, the NEMO approach is based on the Artificial Intelligence of Things (AIoT)—an integration of AI with the physical world. AIoT devices can potentially act as semi-autonomous devices, thus reducing the overall network burden, as well as latency times. NEMO introduces an open-source meta-OS that tries to leverage a variety of novel cutting-edge technologies, such as transfer learning (TL) and FL. The concept of AIoT can be highly beneficial in various time-sensitive applications, such as in the immediate termination of wind turbines in cases of high-speed winds, shutting down machines in latency demanding industrial 4.0 scenarios, and helping autonomous vehicles to avoid fatal collisions.

In the same research direction, the aerOS project [40,41] focuses on the design of a Meta-OS capable of governing the CEI continuum through unified service orchestration, distributed domain federation, and data-centric abstractions. aerOS introduces mechanisms for cross-domain service lifecycle management, enabling applications to be deployed and operated seamlessly across heterogeneous administrative and infrastructure domains. A key contribution of aerOS is the adoption of a Data Fabric approach, which provides a unified and policy-aware data layer across the continuum, facilitating data sharing, interoperability, and trust among distributed components. These functionalities position aerOS as a complementary and integral effort within the Meta-OS research landscape. In [42], the concept of IoT Computing Continuum, or IoTinuum, is introduced. To this end, multiple computing servers are distributed across the IoT continuum to leverage latency-critical calculations. Hence, depending on the application, some sensors may forward data directly to the cloud or others to use the processing nodes. In this context, the IoTinuum is composed of six stages: the S1-thing, which includes all physical layer components, such as sensors and actuators, the S2-Mist stage that includes all low power computing nodes in close proximity to the S1-devices, the S3-Fog stage that includes high computing nodes that can be located far away from sensor nodes, and the S4-Cloudlet that is composed of a reduced set of servers running as a micro datacenter closer to the cloud. Finally, the architectural approach also consists of the S5-Cloud and S6-App, where the later one includes all smart applications. In this context, two use cases are analyzed, with, in particular, smart drone delivery and smart structural monitoring. In [43], the concept of the 6G Computing Continuum (6GCC) is introduced, where a realistic testbed has been used. To this end, a large-scale cell-free AP network is evaluated, for large-scale highly demanding computations.

In [44], a domain-agnostic approach is presented, capable of supporting heterogeneous devices in various network environments. The key advantages of this approach are, among others, a peer-to-peer continuum instead of a hierarchical one, dynamic orchestration of resources, distributed and decentralized learning instead of centralized approaches, and context-aware solutions. To this end, various application scenarios are presented as well, such as smart charging stations for electric vehicles, energy reduction towards carbon-neutral manufacturing processes, just-in-time arrival for vessels, and green driving for reduced fuel consumption and decreased vehicle emission. To this end, the concept of serverless computing is leveraged as well, which is a key concept towards 6G networks. In this context, end-users and developers may describe intents over functions instead of specific hardware components, thus making application development more flexible in terms of privacy preservation and resource engagement.

In [45], a novel DRL approach is discussed, for task offloading in cloud-edge continuum (CEC) systems. Based on this framework, autonomous decisions can be made based on local conditions while dynamically adapting to changing network environments. In this context, task latency and drop rates are optimized, where DRL agents are employed at each edge server. Performance evaluation indicates that significant improvements were achieved compared to baseline methods. As the authors correctly point out, future directions include the extension of this framework to dynamic environments, as well as the creation of a parallel framework to enable fast decision procedures in the IoT devices. The work in [46] presents an optimization framework for smart home energy consumption based on FL. A key novelty of the presented approach is that FL aggregation is not based on simple averaging of the produced models. Instead, newly created data contribute more to the produced global model. The results demonstrate that the proposed method performs similarly or better than other models in terms of prediction error. The last two cited works have been carried out in the context of the EU-funded ICOS project [47].

In [48], a novel concept of self-distributing systems (SDSs) is presented and evaluated. To this end, code mitigation of an application can take place in various resources of the computing continuum. Performance is evaluated against other baseline approaches, as well as with respect to serverless computing. In this context, an agent that is located on top of the proposed four-layered architectural approach performs DRL to extract the optimum subset of resources for code mitigation. As the authors indicate, scalability of this approach, as well as evaluation in more complex scenarios, is a challenging research field for further research.

In [49], a novel symbiotic computing model is introduced, where all participating members in the cloud continuum may share common resources for task improvement. To this end, two approaches are considered, non-cooperative and cooperative resource pricing. In [50], a discussion takes place on containerization and virtualization approaches, in the cloud computing continuum. On one hand, containers can be deployed more easily in lightweight IoT devices; however, they are more vulnerable to security attacks. On the other hand, VMs can offer complete isolation during application execution at the cost of increased hardware requirements. In [51], the problem of optimum planning in multi-area, multi-service, and multi-tier edge cloud computing environments is investigated. To this end, an optimization problem is formulated and solved based on matrix adaptation.

In [52], two novel reference architectures are presented: one for edge–cloud computing models and the other for edge–cloud communication technologies. In the same context, indicative use cases are presented as well. In [53] and in the context of the project NEMO, the open-source framework is described. To this end, various key functionalities, such as security, AI, service and data management, meta-orchestration, and resource management, are provided as open-source components. In [54], security in the continuum is leveraged with the help of physical unclonable functions (PUFs). These kinds of functions are generated using the unique digital identifiers derived from the inherent variability in the manufacturing process of integrated circuits, as a way to enhance security mechanisms at minimal overhead cost [55]. Therefore, in this work a lightweight PUF-generation method is introduced, which can be applied even in cases where only one of the two involved parties can support PUFs. In the same context, a security as a service (SaS) framework is introduced, based on the Chef software (v. 18.9.4) [56].

In [57] the Ratio1, meta-OS is presented. The key features include decentralized ML, as well as device authentication. As the authors point out, large-scale meta-OS deployments along with more advanced privacy policies can trigger further research activities. In [58], an interconnection framework is presented that can leverage the seamless operation of large IoT deployments. This framework uses the Sirius tool and Acceleo, where a smartphone-centric gateway application is used as a mediator to connect devices and sensors within an IoT environment. Finally, in [59], the COGNIFOG concept is presented [60], which is an open-source framework that tries to leverage decentralized ML and decision-making and distributed computing. To this end, container-based virtualization is favored, which is a lightweight and secure alternative that also supports the microservice concept.

All the aforementioned works along with their key directions, supported key driving factors, limitations, and open issues are presented in Table 2 as well.

## 4. Discussion—Open Issues and Key Challenges

From the discussion of the previous section, various open issues and key challenges can be identified in the context of the computing continuum. A key outcome of the presented works is that, although several Meta-OS frameworks have been evaluated through realistic pilots, demonstrators, and open-call experiments within Horizon Europe projects, comprehensive performance evaluation across large-scale, highly heterogeneous, and federated CEI environments remains a challenging issue. More specifically, Meta-OS initiatives such as ICOS, FLUIDOS, NebulOuS, NEMO, and aerOS have demonstrated their architectures in real-world environments, including industrial automation scenarios, smart energy systems, smart cities, and critical infrastructure monitoring. These validations have been conducted through project pilots, as well as open-call experiments, showcasing Meta-OS capabilities in service orchestration, cross-domain federation, data management, and AI-assisted optimization under realistic operational conditions. Notably, these validation activities target vertical sectors that closely match the use cases discussed in Section 5, including Industry 4.0, smart grids, smart cities, and infrastructure monitoring, further reinforcing the practical relevance of Meta-OS architectures in the computing continuum. All approaches so far are based on publicly available datasets or on limited network topologies and testbeds. Another key aspect that was also highlighted is to effectively interconnect a vast number of heterogeneous devices and protocols via secure open access interfaces. The seamless support of highly demanding applications necessitates proper resource management, which is made feasible only via efficient ML approaches. However, the CEI continuum is a highly dynamic environment, where new elements are constantly added/removed from the network; hence, constant updates of the ML models are required. Τo this end and in order to avoid frequent retraining of the derived models, TL can be highly beneficial [61]: the obtained knowledge from another task and dataset (even one not strongly related to the source task or dataset) is transferred to the task under consideration to reduce learning costs. In the same context, the increased number of devices and associated protocols poses new challenges in the design and implementation of efficient security algorithms, since the threat landscape is significantly increased. Hence, ML model training is now a multi-dimensional task, since, apart from resource optimization, the creation of intents for network recovery after attacks is also of utmost importance. However, a trained ML model that provides near-optimum results for the first task (i.e., resource optimization) might not be the ideal one for threat mitigation and vice versa.

Therefore, based on the previous discussion, various key challenges can be identified as well, which are summarized below:

- ML model deployment on lightweight IoT devices. In the CEI continuum, the goal is to offline train an appropriate ML model either at the edge or in the cloud and then deploy it on the IoT device. However, not all IoT devices have the processing capability to run hardware-consuming ML models. Hence, appropriate tiny ML models can be deployed that can effectively run in small IoT devices [62]. In the same context, as previously mentioned, different models may provide near-optimum results for different tasks. In this case, ML repositories in edge and or cloud servers are created and ML model deployment in lightweight IoT devices might occur for more than one trained model, which unavoidably poses additional computational requirements.
- As also mentioned in Section 4, the integration of computing continuum implementations with a 6G architecture is a challenging research field, as it will allow the seamless integration and coexistence of various cutting-edge technologies. However, as the landscape of connected devices increases, security concerns may become a major issue, as previously mentioned. Flexible network architectures allow the identification of multiple types of attacks [63]. To this end, either predictive or mitigation actions can be supported both for well-known and for zero-day attacks, with the help of additional emerging technologies, such as digital twins. In the same context, distributed computing systems are expected to play a key role in this direction, as the deployment of advanced ML algorithms, as well as the support of highly demanding computational applications, such as blockchain technology and encryption [64], cannot be fulfilled by lightweight IoT devices.
- The implementation of the zero-trust context. To this end, constant authentication of all involved devices takes place, which might significantly increase signaling burden in the network.
- When FL is employed for faster ML training times, as well as for privacy protection, a key issue that may rise is non-identical data distribution and severe heterogeneity of the produced datasets (data heterogeneity). This is especially the case in large-scale network orientations with diverse elements. In this case, either subsets of training nodes are formulated, or TL is employed to further improve the ML training latency [65].
- Different policy configurations in various network segments. As stated in the corresponding section, various network and cloud/edge providers may coexist in the computing cloud continuum. In this case, different access and usage policies may pose significant difficulties in proper resource management.
- As the concept of the computing continuum spans across CEI environments, the selection and adaptation of appropriate virtualization technologies remains a challenging issue. Container-based OS-level virtualization offers low overhead and fast deployment, making it attractive for lightweight edge and IoT devices. However, containers provide weaker isolation compared to hardware-level virtualization techniques, such as VMs and lightweight MicroVMs, which are increasingly considered in multi-tenant or security-sensitive edge deployments. Therefore, the challenge is not a binary choice between containerization and virtualization, but rather the improvement and combination of virtualization mechanisms that balance isolation, performance, resource footprint, and orchestration complexity in edge-native environments.
- Related to the last point, emerging execution models such as WebAssembly (Wasm) are gaining attention as lightweight and portable alternatives for workload execution across the computing continuum. Wasm provides strong sandboxing, near-native performance, and fast startup times, making it suitable for constrained edge and IoT environments. In parallel, edge-native orchestration frameworks, such as KubeEdge, extend cloud-native virtualization and orchestration principles toward the edge, enabling consistent management of containerized and virtualized workloads across heterogeneous infrastructures.

**Figure 6:**
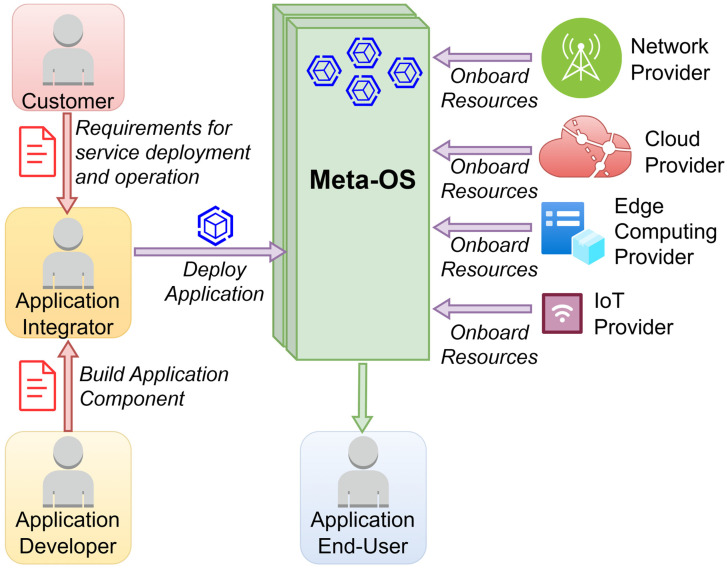
High-level schema of the proposed Meta-OS-enabled computing continuum framework.

Based on the above discussion, a high-level view of a proposed architectural approach is depicted in Figure 6. To this end, various key players can be identified, such as the end user, the network provider, the cloud provider, the edge computing platform provider, the IoT provider, the application developer, and the application integrator. The different roles are analyzed in more detail below:

- Network Provider (NP): The NP is providing the network and connectivity resources that allows the interconnection of the cloud with near-edge and far edge locations, as well as the provisioning of the required resources supporting gateways and remote device connectivity. Within the cloud continuum, there can be multiple NPs depending on the footprint of the infrastructure and the administrative domains.
- Cloud Provider (CP): The CP is provisioning the cloud resources responsible for hosting the application components. Commonly, the CP operates on large cloud infrastructures (e.g., Hyper scalars), providing points of presence (PoPs) of local interest for allowing fast connectivity, low latency, load balancing, and close to the device resource availability.
- Edge Computing Platform Provider (ECPP): Similarly, the ECPP is providing cloud resources at the edge (near or far) of the infrastructure, capable of hosting less resource demanding application’s components coupled with specific hardware (HW) acceleration capabilities suitable for AI/ML workloads. It is assumed that the ECPP infrastructure topology allows for reaching large and/or dense IoT deployments.
- IoT Provider (IoTP): IoT provider is the actor providing the IoT infrastructure that is being deployed across the continuum. This infrastructure may include devices that allow deployment of continuum controllers or/and agents. Moreover, in the case that the capability to deploy the continuum is restricted either due to processing resource limitations or because of access to the HW device OS, appropriate APIs are exploited. The IoTP through the continuum is gaining the ability to open the infrastructure to multiple vertical applications, since all operate on the common continuum software.
- The Application Developer (AppDev) and the Application Integrator (AppInt) can be seen as distinguishing roles played by the same actor or different, depending on the complexity of the ecosystem. The first one is developing application components, enhancing functionality and operation. The latter one is integrating application components that may arrive even from different developers, so that a full-blown application is created and modeled/described in a compatible to the continuum model. In this context the AppInt is experienced with the presented data model, descriptor, and operation specificities. Consequently, the AppDev depends on the Application Integrator to formulate the application descriptors in a way that is comprehensible by the continuum in order to be deployed over an instance.

Hence, as it becomes apparent from the previous discussion, the computing continuum should provide a unified access mechanism to all the aforementioned key players. In the same context, as previously mentioned, a variety of ML models should be trained and updated, either for resource optimization or for threat mitigation. In parallel, security by design should be also supported. Thus, three main modules can be identified, in particular, the Data Management Module (DMM), the Intelligence Module (IM), and the Security Module (SM). The DMM is responsible for managing all data required and exchanged in the continuum, as well as the efficient execution of data-based applications and services used in the continuum. Conceptually, the DMM is aligned with the aerOS Data Fabric paradigm, offering a unified abstraction for seamless data access across the continuum [40]. Building on this concept, the DMM extends it with AI-native data pipelines and dynamic adaptation varying infrastructures. Its main functionalities include the following: (a) data distribution across the continuum, taking advantage of the entire available infrastructure, (b) smart data placement and dynamic adaptation to changes in the infrastructure during operation: devices joining or leaving, reorganizations, etc., (c) Seamless access to data in the continuum, regardless of the location (device or cloud) or nature of the resource (in motion or at-rest), by providing an integrated data platform spanning the whole continuum, and (d) minimization of data transfers to improve performance and trust, by exploiting near-data processing in various types of devices.

The IM provides the functionalities to train, test, use, maintain, and update analytics and ML models in the continuum, with the goal of supporting and augmenting the operations and performance of the security and privacy modules by considering specific policies in the use of data and models, with special emphasis on trustworthiness including the following:

- Intelligence Coordination: Coordination enables optimization and predictive analytics and ML models and its use across the continuum. This will include policies for the use, sharing, and updating of models across the edge-cloud continuum, including FL strategies.
- Data Processing: Data processing and storage in formats and databases optimized for the application of analytics tasks depending on the resources available of the hosting device in the continuum.
- AI Analytics: A library of optimized ML algorithms for the training and testing of predictive and optimization models, including deep learning, adaptive machine learning, and reinforcement learning libraries optimized to operate in constrained devices.
- AI Models Marketplace: A collection of pre-trained analytics and ML models to be reused, updated, refined (e.g., TL), and combined to foster the application of new AI techniques in the different layers of the computing continuum meta-OS. To this end, a challenging task is to provide the functionality to train and compress these models for operation in constrained devices (e.g., pruning unused branches in trees or simplifying NN architectures).
- Trustworthy AI: Provide specific algorithms to analyze the datasets and develop models conforming to policies for privacy and trustworthiness. Functionality for models to be trained in a FL fashion to ensure data protection in datasets containing user-specific data will be provided as well as explainable AI algorithms to provide reassurance of output of models to the different layers of the continuum.

Finally, the SM provides several related functionalities, such as (i) Identity Management; (ii) Authentication, Authorization, and Audit capabilities; (iii) Detection of security issues and mitigation mechanisms (e.g., self-healing); (iv) Support for compliance frameworks; and (v) Trust and privacy.

**Figure 7:**
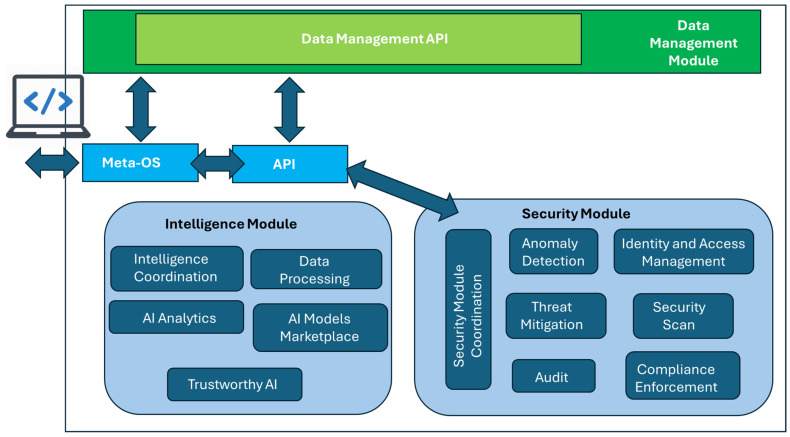
Module placement in the Meta-OS-enabled computing continuum framework.

The diagram shown in Figure 7 illustrates the relationships between the modules of the IM and from the IM to SM, and the DMM. The access point to the IM, as for the DMM, is developed with the help of the appropriate APIs.

## 5. Potential Use Cases

In this subsection, various potential use cases are presented that could be leveraged from the computing continuum and cloud operating systems. These include predictive maintenance in industrial 4.0 applications, load forecasting, smart cities, and critical infrastructure monitoring. For each use case, a reference architecture is discussed along with signaling requirements.

### 5.1. Predictive Maintenance in Industrial 4.0 Scenarios

A challenging use case is the predictive maintenance in advanced industrial 4.0 scenarios, as well as the provision of immediate responses in latency-critical applications. To this end, various IoT devices are placed in key components of the manufacturing process that constantly collect and process data. These data are send to an edge server that is located in the premises of the industry, as shown in Figure 1. To this end, the interconnection of a vast number of heterogeneous hardware components, on one hand, and data analysis for effective ML training, on the other hand, can be made feasible only via the computing continuum. In this case, data analysis targets two actions: (a) predictive maintenance of specific components and (b) immediate termination of a process, in case this is crucial. For example, in a wind park, it is critical for this termination to happen in a predefined time frame, especially in the case of severe wind flows that can damage the machine. In the same context, these edge servers that collect local data can train ML models, as previously mentioned. However, in the case of similar industrial premises, on-site edge servers can locally train an ML model, which can be aggregated in a FL fashion way after collecting all parameters from all premises. Although FL ensures privacy by design since only model parameters are exchanged, communication among the premises can be leveraged with the use of private networks [66].

### 5.2. Load Forecasting in Smart Grid Environments

In load forecasting in the smart grid energy sector the goal is to collect data from various IoT sensors spread across the grid topology and perform accurate load forecasting. These sensors can be placed in production units, renewable energy sources, and in households. The aim is to periodically send data to edge servers that collect them and perform accurate load forecasting. As in the previous scenario of predictive maintenance, the goal is to securely interconnect a vast number of interconnected devices that can be spread over large geographical areas. In advanced scenarios, household energy consumption can be measured via IoT measurement devices where, with the use of appropriate applications, a user can be informed on the actual amount of data under consumption, as well as on the predicted data in predefined time intervals. Depending on the latency tolerance, ML model training can take place either on local edge servers or in the cloud domain, especially in cases of long-term production planning.

**Figure 8:**
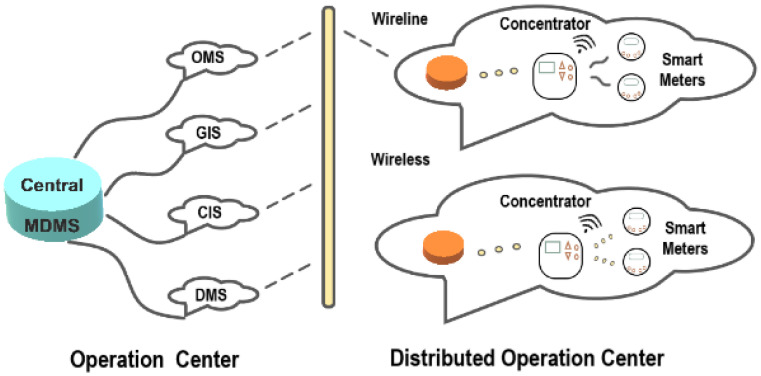
A dispersed communication structural design for Smart Grid based on IoT.

A typical use case, for example, is depicted in Figure 8, where concentrators collect data generated from smart meters in households. In the operation center, proper IoT data are collected from various modules, such as the geographical information system (GIS) that matches consumed energy with specific households, the data management system (DMS) for load forecasting, the consumer information system (CIS) that provides billing information, and the operational management system (OMS) that informs the central management system for potential outages.

### 5.3. Smart Cities

In the concept of smart cities, the goal is to place various IoT sensors and measure key performance metrics, such as traffic, air quality, people density, etc. [65]. Hence, data generated from a variety of sensors should be properly collected to optimize various parameters directly related to the well-being of citizens, such as the time of arrival of transportation, management of traffic in roads, light density, etc. This concept is also applied in smart buildings, where various operations, such as central heating and cooling, air quality, etc., are managed by on-site edge servers [67,68]. As it becomes apparent from the above, a full deployment of the smart city concept presumes the integration of multiple IoT heterogeneous sensors. Hence, data management, communication procedures, and a data integration for efficient ML model training can be quite challenging tasks. To this end, a full layered approach as the one presented in Figure 1 would be the best choice for this scenario. Such an approach can also leverage the deployment of advanced services and applications in 6G scenarios within smart cities, such as autonomous driving.

### 5.4. Infrastructure Monitoring

In this use case scenario, various IoT sensors are deployed over large-scale infrastructures to collect data and leverage predictive maintenance. In railway lines for example, the massive deployment of sensors along different parts of the infrastructure is essential for the optimization and improvement of service and safety. The increasing number of sensors and its specific and typically siloed solutions present an increasing complexity related to the management and operations of such solutions.

Today, the railway-monitoring process to improve the maintenance cycle is basic, and for most railway operators, it is performed preventively (once every fixed period) through a special train with sensors on its wheels, which runs through the whole rail system. This special train can measure several key parameters of the railway system, such as the height difference and width between two lines and thus identify where, potentially, corrections in the lines are needed. However, this measurement is only taken every once or twice a year; in the remaining months, nobody knows what happens (only physical inspections are available: very costly and uncommon), and moreover, there is no established procedure to evaluate the cost-effectiveness of the actions taken to address the identified rail line issues. In this context, digital technology, such as the IoT, aims to minimize the monitoring and maintenance costs by gaining knowledge of the status of key aspects of the railway infrastructure in real-time: rail tracks levelling, tensions, and slope, surrounding areas settlements and falling elements, catenaries maintenance, cyber processes monitoring, etc.

Hence, the main challenge to be addressed by this use case is related to the continuous monitoring of critical infrastructure on rail tracks to ensure safety and improve maintenance activities. In the same context, energy-efficient solutions for low-power IoT devices are required to guarantee safety operation monitoring in real time while ensuring a very long lifetime of the deployed technology in remote locations. The aforementioned concept can be applied in additional large-scale infrastructures as well. Two typical examples include large hydroelectric stations, where various sensors are deployed over the topology to measure key performance indicators, (e.g., water flow, resistance of the barrier, water quality, etc.) as well as in large bridge infrastructures. In the latter case, cracks or malfunctionalities throughout the construction can be easily identified.

**Figure 9:**
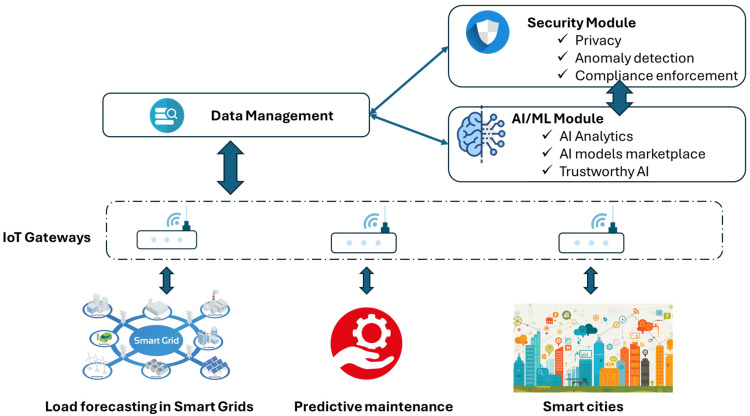
Use cases for interactions and data exchange in the meta-OS-enabled and AI-assisted computing continuum.

A schematic overview of data exchange for various applications in the meta-OS computing continuum is shown in Figure 9. To this end, multiple IoT gateways gather related information from the physical sources and forward it to the continuum instances. For ease of abstraction, the DMM, AI/ML, and the SM can be placed in an arbitrary module of the continuum, as introduced in Figure 1. Once data is received by the DMM, both the SM and the IM can process this information and either update the corresponding ML models or enforce threat detection and privacy policies. In this context, the compliance-enforcement module will enable the detection of compliance problems and enforce infrastructural changes in order to enhance compliance based on the chosen target compliance framework. The privacy module will provide a set of primitives that could be used for data transformation, such as anonymization, and encryption. The anomaly-detection module either receives updated ML models from the IM for threat detection or forwards corresponding data in cases of zero-day attacks.

Beyond infrastructure monitoring, this use case also highlights the importance of observability at the Meta-OS level. In greater detail, the Meta-OS should provide native support for continuous observability of the computing continuum, enabling the collection, correlation, and analysis of system-level telemetry (metrics, logs, traces, data flows, and AI model behavior), comprising IoT devices, gateways, edge, and cloud resources. In this manner, the Meta-OS will be able to evaluate the health, performance, energy efficiency, security level, and compliance of the continuum in a timely manner. This information can be exploited by the DMM, IM, and SM to support closed-loop automation, including adaptive data placement, proactive fault detection, dynamic reconfiguration, and trustworthy AI operation.

## 6. Conclusions

In this article, all recent technological developments in the area of meta-OSs in the computing continuum were analyzed, with an emphasis on the support of advanced services and applications. In the same context, several open issues and limitations were identified as well. As it becomes apparent, various key enabling technologies will support this context, such as advanced machine learning algorithms that properly collect data and perform large scale distributed optimization, as well as efficient and lightweight security mechanisms. However, as it became apparent from the discussion in this article, all adopted solutions should be based on open-access frameworks, to facilitate interoperability of the connected devices and leverage scalability. In the same context, coexistence with the 6G networks is also a challenging issue, as IoT, edge, and cloud devices should be in the position to support advanced services and applications.

Hence, a cutting-edge research effort lies in the deployment of advanced ML approaches that can rapidly adapt to varying network conditions, leverage green computing calculations, and, at the same time, minimize intent creation times for various threats. In this context, a high-level view of a proposed architectural approach was discussed as well that facilitates the entry and communication of various entities of the continuum and, at the same time, leverages the creation and storage of advanced ML algorithms to be adopted per case.

## Figures and Tables

**Table 1:** Indicative related surveys.

Paper	Year	Key Directions	Limitations and Open Issues
[5]	2023	Cloud-Edge-IoT Systems	Focus is on CEI systems and not on access or data management architectures in the continuum
[13]	2023	Cloud Edge orchestration at the edge	Evaluation of containerization or virtualization in real world scenarios
[15]	2024	AI on the edge	Cloud-Edge orchestration Hardware integration to support advanced AI/ML applications
[16]	2023	Architecture of distributed computing systems	Learning Models Intelligent protocols for effective resource management
[17]	2025	Recent trends in computing continuum systems	Flexible resource allocation Mobility in the continuum
[18]	2025	SDN and NFV in cloud edge orientations	Performance evaluation in real-world orientations
Our work	-	Architectural approaches of meta-OSs for the computing continuum	-

**Table 2:** Related works.

Paper	Year	Key Directions	Key Driving Factors				Limitations and Open Issues
			AI/ML	Security	6G	Open Access	
[34]	2024	Presentation of the FLUIDOS Project AI optimization during application execution	×				Deployment in real-world scenarios
[36]	2023	Presentation of the NebulOus project	×	×			Performance evaluation in real world scenarios
[38]	2024	Presentation of the NEMO Project Open-source components for various features (e.g., AI, security, service and data management)	×	×		×	Performance evaluation in large scale scenarios
[40]	2025	aerOS Meta-OS; cross-domain service orchestration; distributed domain federation; Data Fabric					Large-scale validation across heterogeneous administrative domains
[42]	2024	Six proposed stages of the IoT Computing Continuum	×				Integration of programmable network stages
[43]	2022	6G Computing Continuum			×		Integration of the computing continuum with 6G architectural approaches
[44]	2022	Presentation of the RAMOS concept	×	×	×	×	Context-aware machine learning
[45]	2024	Task offloading in IoT Cloud Edge scenarios via DRL	×	×		×	Extension in dynamic topologies Additional performance metrics during optimization
[46]	2023	Federated learning in IoT scenarios	×	×		×	Evaluation in additional real-world scenarios
[48]	2023	Application resources distribution in the computing continuum	×				Evaluation of the SDS approach in more complex scenarios Scalability
[49]	2025	Resource pricing in computing continuum					More diverse user behavior scenarios
[50]	2024	Virtualization vs. Containerization in the cloud continuum	-	-	-	-	Performance evaluation of bigger hardware architectures for Edge or Cloud Security issues in both approaches
[51]	2025	Edge–Cloud Continuum Planning	×				Integration of AI techniques
[52]	2024	Edge cloud computing and communication	×	×			Efficient communication technologies for the different parts of the continuum
[53]	2024	Open-source framework of NEMO project	×	×		×	Performance evaluation in large scale scenarios
[54]	2023	Physical Unclonable Functions		×			Evaluation in realistic scenarios
[57]	2025	Ratio1 meta-OS Decentralized ML and device authentication	×	×			Additional privacy policies Broader cross-chain interoperability
[58]	2023	Large scale interconnection of IoT devices	-	-	-	-	Only one smartphone was used for performance evaluation Additional testing with diverse IoT devices
[59]	2025	The COGNIFOG framework	×	×		×	Orchestration intelligence Decentralized, privacy-preserving AI training at the edge

## Data Availability

Non applicable.

## Abbreviations

The following abbreviations are used in this manuscript:

5G	Fifth Generation
6G	Sixth Generation
6GCC	6G Computing Continuum (6GCC)
AI	Artificial Intelligence
AIoT	Artificial Intelligence of Things (AIoT)
AP	Access Point
API	Application Programming Interface
AppDev	Application Developer
AppInt	Application Integrator
CEC	Cloud Edge Continuum
CEI	Cloud Edge IoT
CIS	Customer Information System
CP	Cloud Provider
DCS	Distributed Computing System
DMM	Data Management Module
DMS	Data Management System
DRL	Deep Reinforcement Learning
ECPP	Edge Cloud Platform Provider
EU	European Union
GIS	Geographical Information System
FL	Federated Learning
HW	Hardware
IM	Intelligence Module
IoT	Internet of Things
IoTinuum	IoT Computing Continuum
IoTP	IoT Provider
ML	Machine Learning
MQTT	Message Queuing Telemetry Transport
NFV	Network Function Virtualization
NN	Neural Network
NP	Network Provider
OS	Operating System
RL	Reinforcement Learning
PaaS	Platform as a Service
PoP	Point of Presence
PUF	Physical Unclonable Function
QoS	Quality of Service
SaS	Security as a Service
SBA	Service Based Architecture
SDN	Software Defined Networking
SDS	Self-Distribution Systems
TL	Transfer Learning
Wasm	WebAssembly
